# Management Practices for Hemodynamic Impairment in Neonates Born Prematurely: A Quality Improvement Project

**DOI:** 10.3390/jcm13226848

**Published:** 2024-11-14

**Authors:** Océane Lalin, Simona Gaga, Jean-Michel Hascoet

**Affiliations:** 1 Neonatology Department, Maternité Régionale Universitaire—CHRU Nancy, 54000 Nancy, France; s.gaga@chru-nancy.fr (S.G.); j.hascoet@chru-nancy.fr (J.-M.H.); 2 DevAH 3450, University of Lorraine, 54000 Nancy, France

**Keywords:** preterm neonates, hemodynamic impairment, quality of practice, echocardiography

## Abstract

**Background:** No consensus exists on the management of hemodynamic impairment in very premature neonates. At level 3 NICU, the protocol involves an initial infusion of crystalloids, followed by a cardiac ultrasound if the infusion fails to restore appropriate hemodynamics. Based on the ultrasound findings, a decision is then made regarding a second infusion or the prescription of vasopressor amines. The aim of the present study was to assess the effect of and compliance with this management practice in neonates born prematurely between 26 and 31 completed weeks of gestation following a plan-do-study-act design. **Methods:** Data were collected retrospectively from patient records for all neonates who were managed for hemodynamic impairment within the first 24 h of life. **Results:** Of 604 neonates born during the study period, 68 were included in this study, but only eight cases followed the protocol. Reasons for non-compliance were the absence of cardiac ultrasound and variations in the duration and dosage of fluid administration. There was a significant relationship between blood pressure and positive inspiratory pressure levels at the time of management and compliance with the protocol. **Conclusions:** A revision of the protocol will emphasize the importance of echocardiography assessment, as all neonates responded to the ultrasound-guided therapy. As a quality improvement measure, attending neonatologists will be trained to thoroughly adhere to the protocol before the next evaluation.

## 1. Introduction

The prevalence of preterm birth is approximately 8% among all births in France [1]. Health issues related to preterm birth are the main cause of death among children under 5 years of age, including hemodynamic disorders. Management of hemodynamic impairment during the first days of life is currently a challenge, especially in neonates born prematurely between 26 and 31 completed weeks of gestation, for which the most appropriate care is not well-defined [1,2,3].

During development, circulation evolves in three steps: fetal circulation; early neonatal circulation; and final postnatal circulation. At birth, the first breathing movements increase pressure and flow in the left atrium by increasing pulmonary output. The last step occurs after the three shunts close, resulting in circulation splitting into two circuits that flow in series: pulmonary circulation and systemic circulation. During this period, the left ventricle adapts to the higher afterload by thickening its walls [4,5,6]. In preterm neonates, the lung’s capillary surface is reduced, leading to pulmonary hypertension. Additionally, the immature myocardium results in impaired contractility and compliance compared to infants born at term [7]. Furthermore, immaturity of the cardiorespiratory system increases the risk of hypotension and shock. The definition of hypotension in this population remains controversial and includes blood pressure lower than the 10th percentile for birthweight and age, mean blood pressure < 28–30 mmHg, or mean blood pressure lower than the gestational age of the patient in weeks [8].

Leading causes of low blood pressure in preterm infants include functional adrenal insufficiency [9,10], infection, cardiopathy, and iatrogenic factors such as sedation, maternal therapy, or artificial ventilation [11]. Hypotension with impaired hemodynamic status can lead to brain or intestinal damage [4]. However, with appropriate tissue perfusion, it may be temporarily well tolerated and harmless, a condition referred to as “permissive hypotension” [12]. In routine care, it is often challenging to differentiate between these two situations and determine the appropriate therapy during this transitional period. A multiparametric assessment is necessary to evaluate hemodynamic status [13]. This assessment includes clinical, biological (serum lactate levels, biological organ function), and echocardiographic parameters (blood volume, cardiac output, myocardial contractility) [14]. Clinical signs of hemodynamic impairment include low diuresis, high heart rate, high capillary refill time, and low blood pressure [4,8,12]. Although no validated recommendation exists for treating hemodynamic disorders, options include fluid infusion, vasoactive agents, or corticosteroids.

Due to advances in neonatal care and medical techniques, more neonatologists have acquired skills in echography, particularly in functional echocardiography. This makes it possible for a neonatologist to perform a simple hemodynamic assessment at any time. Functional echocardiography in neonates focuses on assessing myocardial function and blood flow rather than morphological evaluation, which is the domain of pediatric echocardiography [15,16,17,18].

Vasoactive drugs can act on different receptors, making it crucial to understand the underlying physiopathology when choosing the appropriate therapy. In cases of adrenal insufficiency, hydrocortisone can be used [19]. Additionally, prenatal corticosteroids, optimal ventilation, and sedation are effective measures to prevent low blood pressure with reduced organ perfusion.

**Figure 1:**
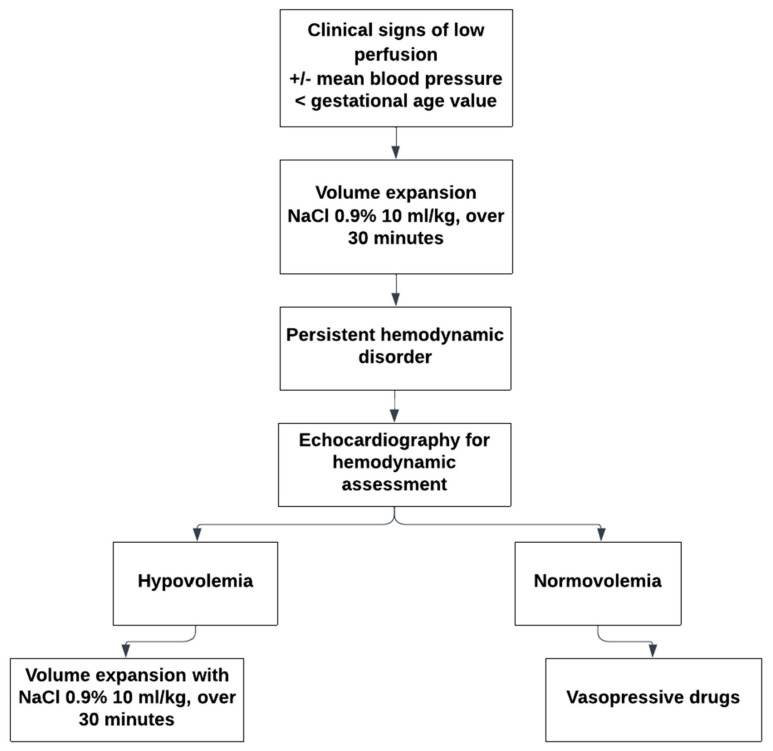
Current protocol for the management of hemodynamic disorder in the Regional University Maternity Level 3 Hospital.

At the regional university, level 3 neonatal intensive care unit (NICU) of the maternity hospital in Nancy, a pragmatic protocol defines low blood pressure as a mean blood pressure lower than the infant’s gestational age in weeks. In case of clinical signs of low perfusion associated with low blood pressure, the protocol advises initially administering crystalloids (10 mL/kg of saline solution over 30 min) or blood products if anemia is also present. If fluid infusion does not improve the clinical status, functional echocardiography is indicated to assess the hemodynamic status of the infant. Depending on the etiology of the hemodynamic failure, vasopressive drugs may be indicated. For preterm babies who have not received prenatal steroid maturation, hydrocortisone may be prescribed (Figure 1).

Given the various etiologies of hemodynamic disorders in preterm infants, each requiring specific therapy, and the lack of widely accepted guidelines, we decided to undertake a quality improvement project. This project aimed to assess current practices for managing hemodynamic disorders within the first day of life in neonates born prematurely between 26 and 31 completed weeks of gestation. The primary outcome measure was to evaluate compliance with the protocol and the reasons for non-compliance. A secondary outcome measure was an evaluation of the protocol’s effect on hemodynamic stabilization.

## 2. Materials and Methods

### 2.1. Type of Study

This was an observational, retrospective, single-center cohort study analyzing practices in a level 3 NICU. This study was organized as a Plan-Do-Study-Act (PDSA) project: assess compliance with our pragmatic protocol and identify causes of non-compliance; collect data from the infants’ records; evaluate and analyze the reasons for non-compliance; and propose appropriate revisions to the protocol to complete the cycle.

### 2.2. Study Population

**Figure 2:**
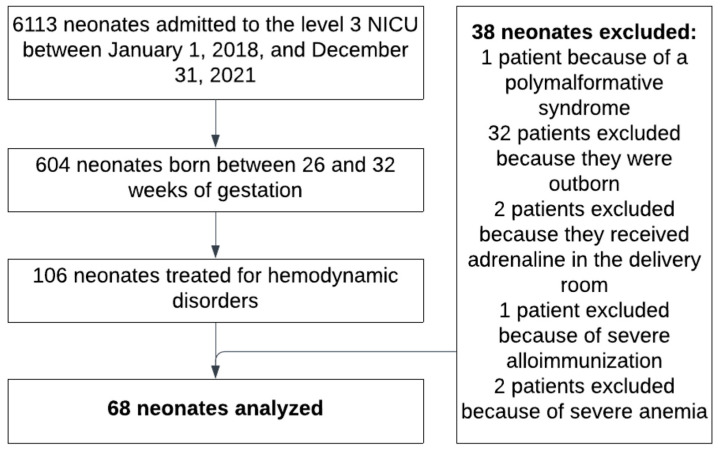
Flow chart of study inclusion.

All newborns born between 26 and 32 weeks of gestation who were admitted to the level 3 NICU between 1 January 2018 and 31 December 2021 and received therapeutic management for hemodynamic disorder within the first 24 h of life were included in this study. Exclusion criteria were being born in other maternity units and secondarily transferred, any genetic disorder or malformation, presenting with severe anemia or massive hemorrhage, or receiving adrenaline treatment in the delivery room (Figure 2).

### 2.3. Data Collection

The list of infants born prematurely between 26 and 31 completed weeks of gestation during the study period was obtained from the Medical Information Department of Nancy University Hospital. A table of correspondence (infant’s name, date of birth, and anonymity number) was established and kept secure in an appropriate place (Office of the Head of Department). Only the children’s anonymity numbers appeared on the computer files created for this study (i.e., data collection, processing, and production of results). Data were collected from the hospitalization records, both computerized and paper. Data concerning diuresis and drug prescriptions were recorded on paper charts until December 2021.

Collected variables included information on the pregnancy, such as gestational diabetes, pre-eclampsia, infectious risk factors, maternal treatments and history, antenatal maturation, and smoking or drug use by the mother. Data were also collected on birth conditions, including Apgar score, gestational age, birth weight, delivery method, pH, lactate level, and diuresis, as well as on management in the delivery room, such as ventilation mode and drug administration. Additionally, data on early management in the NICU were recorded, including treatments and hemoglobin and hematocrit levels at admission. A pre- and post-treatment evaluation was conducted by collecting data every 30 min during the 4 h before and after hemodynamic management. Diuresis and echocardiographic values were recorded both before and after treatment. Data related to fluid management—such as the timing, duration, and type of fluid administered, as well as the use of vasoactive amines—were also recorded.

The success or failure of the treatment to normalize hemodynamic parameters was recorded. When the first treatment failed, the second and third treatments were evaluated.

Individuals’ current treatments were compared with the planned protocol. In the case of non-compliance with the protocol, the reason was noted.

We also collected information on short-term outcomes of the presence of a ductus arteriosus up to the third day of life. Similarly, we analyzed morbi-mortality, including bronchopulmonary dysplasia, retinopathy of prematurity, necrotizing enterocolitis, intraventricular hemorrhage, and death. Bronchopulmonary dysplasia was defined as the need for oxygen after 36 weeks postmenstrual age.

Our Institutional Review Board approved this study (Delegation à la Recherche Clinique et à l’Innovation du CHRU de Nancy (DRCI number: EPP2022-006)). Parents provided written consent for their infant’s data to be used for research purposes.

### 2.4. Statistical Analysis

Categorical data are presented as numbers or percentages. Assuming that successful compliance with the protocol would be 80% success, a sample size of 68 infants with alpha = 0.05 would lead to a power of 0.80. Chi-squared was used to evaluate the differences between groups for categorical variables. Continuous variables that were not normally distributed are presented as medians with the interquartile range (IQR) and were analyzed using the Mann–Whitney U test. The parameters significantly influencing compliance in the bivariate analysis and the factors responsible for non-compliance were analyzed by multivariate analysis. A *p*-value < 0.05 was considered significant. All analyses were performed in SYSTAT 13 software (2007, Systat Software Inc., San Jose, CA, USA).

## 3. Results

### 3.1. Main Demographic and Baseline Characteristics

A total of 6113 neonates were admitted to the NICU of Nancy Maternity Hospital between January 2018 and December 2021. Of these, 604 were born prematurely between 26 and 32 weeks of gestation, and 106 received hemodynamic treatment within the first 24 h of life. Thirty-eight neonates were excluded from this study based on the exclusion criteria (Figure 2). Finally, 68 neonates (61.8% male) with a median gestational age of 28 weeks (IQR 27–29) were included in this study. The birth weight ranged from 450 g to 1705 g, with a median weight of 965 g (IQR 839–1200).

Among the 68 infants included in this study, 36.7% were born in a context of spontaneous prematurity and 63.2% after induced prematurity; 54.4% were eventually born vaginally and 45.6% by Caesarean section. A risk of maternofetal infection was present in 66% of the infants, and 73.1% were fully matured by antenatal corticosteroids. The median Apgar score was 3 at 1 min of life and 6 at 5 min of life. All infants were intubated in the delivery room. Among the neonates included in this study, 69.1% received antibiotic treatment prior to the hemodynamic disturbances, and 97% received analgesic treatment prior to the hemodynamic disturbances. Birth conditions and pregnancy characteristics did not significantly influence compliance with the protocol (Table 1).

Group 1 was defined by compliance with the protocol and group 2 by non-compliance with the protocol. There was no significant difference between the two groups in regard to birth weight [median 1000 g (IQR 887–1070) in group 1 versus 955 g (IQR 844.5–1234) in group 2; *p* = 0.924] or gestational age [median 27 weeks (IQR 27–29) in group 1 versus 28 weeks (27–28.25) in group 2; *p* = 0.977]. Group 1 was 62.5% male, and group 2 was 61% male (*p* = 0.964).

### 3.2. Parameters

**Figure 3:**
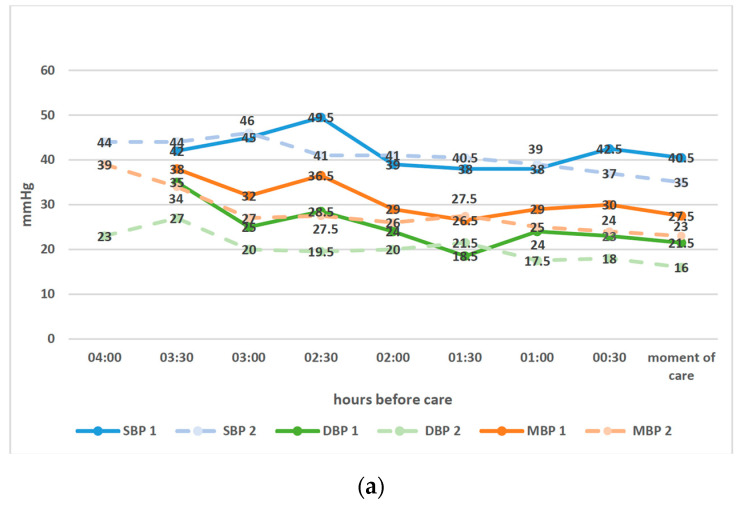

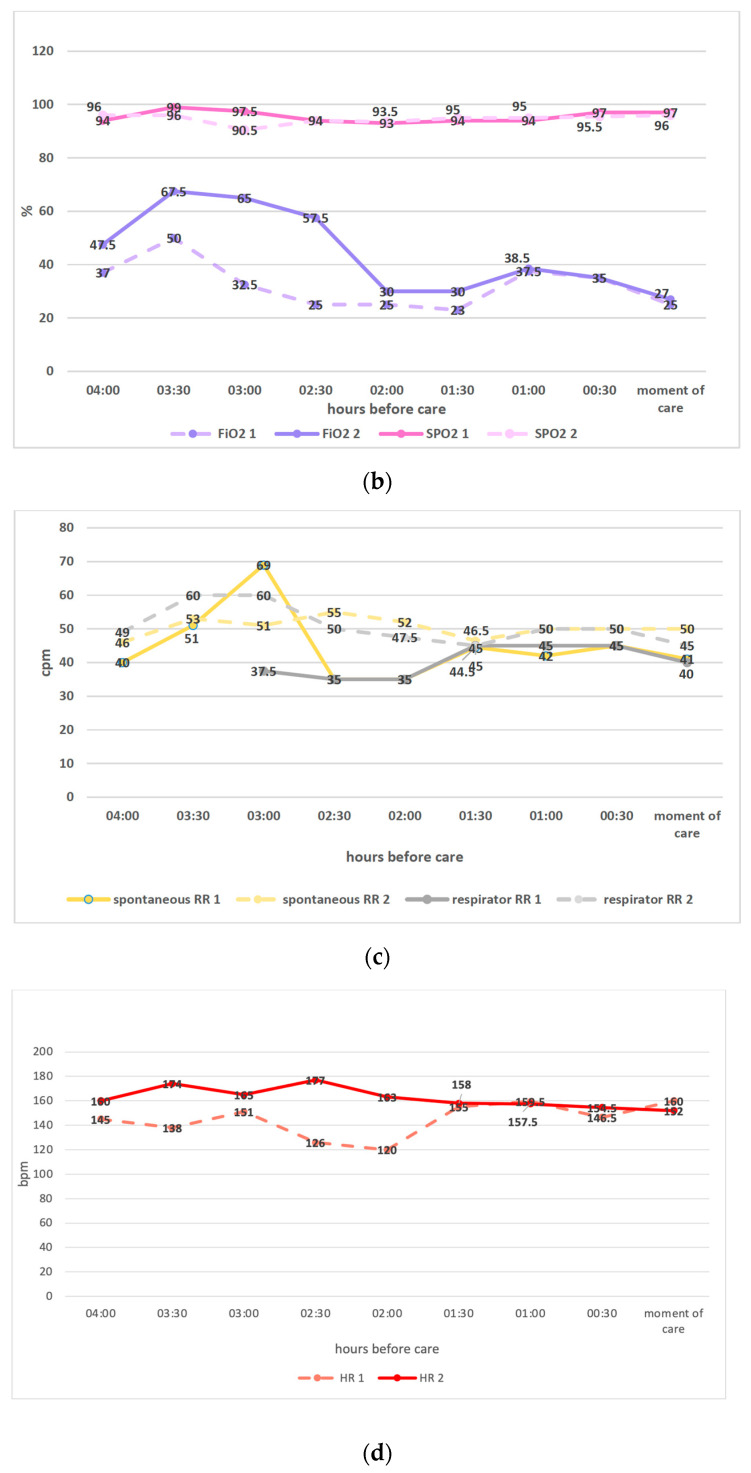
Parameters prior to care according to adherence to the protocol. (**a**) Mean blood pressure before care (first hemodynamic management). (1) infants compliant with the protocol; (2) infants non-compliant with the protocol. SBP, systolic blood pressure; (**b**) Mean SpO2 and FiO2 before care (first hemodynamic management). (**c**) Mean RR before care (first hemodynamic management). (**d**) Mean HR before care (first hemodynamic management). HR, heart rate; bpm, beats per minute; RR, respiratory rate; cpm, cycle per minute; DBP, diastolic blood pressure; MBP, mean blood pressure.

Among all parameters collected prior to hemodynamic management, the mean blood pressure at the time of management was 27.5 mmHg (IQR 26–28) in group 1 and 23 mmHg (IQR 20–25) in group 2 (*p* = 0.007). The diastolic blood pressure at the time of management was a median of 21.5 mmHg (IQR 20–24) in group 1 and 16 mmHg (IQR 13–19) in group 2 (*p* = 0.001). Peak inspiratory pressure was a median of 19 cmH20 (IQR 18–20) in group 1 and 20 cmH20 (IQR 19–21) in group 2 at the time of management (*p* = 0.037). These variables were the only variables with a significant impact on protocol compliance (Figure 3). The respect of the indication to receive treatment was also a significant factor in good compliance with the protocol, as 83% were treated appropriately (*p* = 0.03).

### 3.3. Management of Hemodynamic Disorder

**Figure 4:**
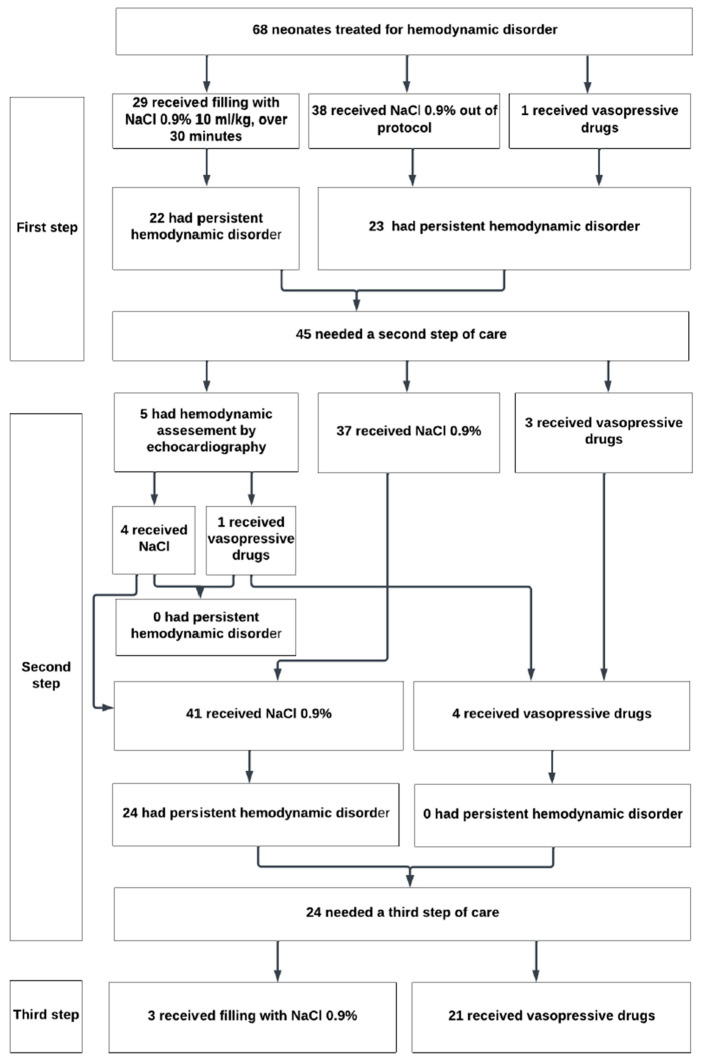
Actual care received by the neonates within the first 24 h of life.

The onset of treatment occurred between 20 and 540 min of life [median 107 min (IQR 80–150)]. In 98% of cases, the initial hemodynamic management consisted of volume expansion as specified in the protocol. Volume expansion was always performed with NaCl 0.9% for a duration of 20–120 min (protocol indication: 10 mL/kg over 30 min). For 34 neonates, the reason for non-compliance was the rate of infusion; 10 received the infusion faster than the 30 min indicated in the protocol, and 24 received the infusion for a longer duration. Dosing varied from 10 to 20 mL/kg. In 91% of neonates with persistent hemodynamic impairment, the second approach to hemodynamic management consisted of additional volume expansion. Only 5/68 neonates underwent hemodynamic assessment by echocardiography as indicated in the protocol to justify the treatment being used (Figure 4).

Among the included neonates, 83% received appropriate hemodynamic management because of a low mean arterial blood pressure with signs of impaired perfusion. The other neonates had a severe clinical status but no sign of impaired hemodynamic function, as stated in the protocol. Regarding secondary outcomes, of the 68 infants included in this study, 27% had a patent ductus arteriosus on the third day of life; 42% presented with bronchopulmonary dysplasia; 29% were followed for retinopathy of prematurity, 21% for intraventricular hemorrhage, and 13% had necrotizing enterocolitis. Among the infants in group 1, 50% suffered from retinopathy (all stages); 25% were treated for patent ductus arteriosus; 37.5% suffered from bronchopulmonary dysplasia; 25% had necrotizing enterocolitis, and 12.5% were diagnosed with intraventricular hemorrhage. Among the included infants, 62 survived, but there was no significant relationship between survival and compliance.

### 3.4. Multivariate Analysis

Echocardiography, before management and filling duration, was the factor independently involved in non-compliance (Table 2).

## 4. Discussion

The aim of this quality improvement project was to evaluate the management of hemodynamic disorders by assessing the compliance with our NICU protocol within the first 24 h of life and to look for perinatal factors influencing this compliance. The protocol was only appropriately followed for 8 of the 68 (12%) neonates included in this study. The main reason for the lack of compliance was the absence of functional echocardiography for hemodynamic purposes in 40/45 (89%) neonates after the failure of the first treatment. Despite four attending neonatologists trained and certified in echocardiography, the clinicians did not feel that this evaluation was important.

Our study analyzes the adherence to and effectiveness of a local protocol, written and applied solely by the neonatal intensive care unit of CHRU Nancy. In the literature, there are no official recommendations for managing hemodynamic disorders in preterm infants. As suggested by the study by Mullaly et al. [20], it is recommended to perform hemodynamic ultrasound in cases of suspected low flow based on clinical and biological criteria, followed by therapy that will depend on the echocardiography results. In cases of decreased systemic vascular resistance, a vasopressor treatment will be indicated; if there is impaired cardiac output, the team suggests introducing inotropic treatment. Finally, in cases of vasoplegia, restoring volume status through volume expansion and possibly introducing a vasopressor and hydrocortisone treatment will be necessary.

Echocardiography is typically not included in the routine assessment of hemodynamic disorders in neonatology, which generally involves clinical assessments of blood pressure, heart rate, capillary refill time, and diuresis, as well as biological assessments of lactate levels. However, by using functional echocardiography, we can easily determine whether there is sustained hypovolemia and/or a defect in cardiac contractility by measuring left ventricular ejection fraction (LVEF), cardiac output, and systemic vascular resistance. This approach allows for the rapid initiation of effective treatment without resorting to probabilistic therapy, which can be harmful.

In our study, 8 out of 8 neonates who underwent echocardiography had successful treatment outcomes after the second step, compared to 5 out of 40 without echocardiography. The success rate with echocardiography across all steps was 100%, whereas without echocardiography, the success rate was 60%. This suggests that echocardiography is particularly useful after the failure of initial treatment and should be considered mandatory. However, it is important to note that the small sample size in this study limits the strength of these conclusions. We cannot rule out the possibility that some second treatments might have failed despite echocardiography if more infants had been included.

Notably, Pugnaloni et al. [21] suggested that neonatologist-performed echocardiography could be beneficial in cases of septic shock and emphasized the need to use functional echocardiography to guide hemodynamics-based treatment strategies.

Another factor contributing to non-compliance with the protocol was the duration of volume expansion. Our current protocol recommends volume expansion over 30 min, but this was achieved in only 50% of the infants who received volume expansion. Ten infants received the infusion faster than the 30 min indicated in the protocol, and 24 received the infusion for a longer duration. The cut-off time of 30 min was chosen arbitrarily. The aim of volume expansion is to increase the preload. Therefore, the duration of volume expansion should not be too long. It should also not endanger the venous capital, which can be precarious in premature infants and was the reason given. There is currently no consensus on this subject. Bark et al. [22] showed a lack of significant difference in efficacy between administering NaCl 0.9% over 15 min versus 3 h. Although this was not the primary aim of our study, it does seem to support the actual practice. There was no greater failure in newborns who received NaCl 0.9% over 30 min compared with 15 min. Therefore, a revised protocol could suggest an interval of 15 to 60 min.

The dosing of volume expansion was also a factor of non-compliance with the protocol in bivariate analysis but not an independent factor in multivariate analysis. The protocol suggests the administration of 10 mL/kg NaCl, as the first dose is administered blind to echocardiography. The chosen volume represents a filling test to assess its efficacy while limiting adverse effects. The aim of volume expansion is to obtain an increase in preload while limiting the increase in pressure in the venous vascular bed as much as possible, which would accentuate capillary leakage. As described in Weaver’s study [23], excess fluid intake, including excessive volume expansion, may be deleterious, with a risk of acute pulmonary edema. To assess the response to filling, hepatic compression could be considered to evaluate the effectiveness of increasing the preload without being deleterious, in case the hemodynamic disorder was not linked to a volume defect. Notably, 17% of the infants received hemodynamic management even though they did not meet the protocol’s treatment criteria. Thus, they did not present clinical signs of hypoperfusion in association with low blood pressure, or the blood pressure increased before beginning the infusion.

Numerous studies have looked at the right time for hemodynamic management, and a common conclusion is that arterial pressure should not be taken into consideration alone. It is important to look for clinical or paraclinical signs of low cardiac output. Binder-Heschl et al. [24] showed that moderate hypotension of short duration did not influence cerebral vascularization because an autoregulatory system protected the newborns. Carrapato et al. [14] concluded that we must not rely solely on blood pressure but consider the various clinical and paraclinical elements at our disposal to assess hemodynamic impairment. Permissive hypotension in the transient phase can be accepted if it is hemodynamically well-tolerated. In contrast, hypovolemia leads to a reduction in venous return to the heart. The resulting tissue hypoperfusion and cellular hypoxia are due to reduced arterial oxygen transport. Therefore, hemodynamic assessment must consider several factors, such as mean arterial pressure and organ perfusion, including diuresis, heart rate, oxygen saturation, and consciousness.

In our protocol, volume expansion is performed with 0.9% NaCl. A complication may be hyperchloremic acidosis. Indeed, a non-neutral acid-base balance is a risk factor for amine inefficiency. Hyperchloremic acidosis can also lead to long-term renal failure. Numerous studies [25] have looked into the solute of choice for volemic expansion, but there is no official recommendation. The composition of the solution and any pre-existing ionic disorders in the newborn should be considered when choosing the most suitable solution.

Our study has strengths and limitations. One strength of this study is that it is a cohort study covering the entire population, with approximately 10% of neonates born prematurely between 26 and 31 weeks of gestation affected. However, this study has limitations. It is a retrospective study conducted at a single center, meaning that protocols may vary in other neonatal intensive care units (NICUs). Additionally, measurements may not have been very precise if they were not taken correctly (e.g., if a cuff was not properly adjusted to the infant’s size), which cannot be assessed retrospectively. Not all infants underwent hemodynamic assessment, but only those with clinical signs of impairment were assessed, which may represent a selection bias. However, these infants would not be included in the management protocol regardless. Therefore, this will not modify the results of this study. Finally, the population of the protocol-compliant group was too small to draw valid conclusions regarding comorbidities.

## 5. Conclusions

The primary factor contributing to non-compliance with our protocol was the omission of echocardiography before initiating management of the hemodynamic disorder. Our study supports the significant value of echocardiographic assessment when combined with clinical and biological evaluations to achieve appropriate management without adverse effects. Consequently, an ongoing training strategy for hemodynamic echocardiography for all attending neonatologists in the department appears to be a worthwhile initiative.

It seems important that the department’s new protocol should emphasize the need for signs of hemodynamic impairment before starting treatment. Management will then depend on all of the clinical and paraclinical data, resulting in treatment with a vasopressor or inotropic therapy or volemic expansion (20 mL/kg over 15–60 min), depending on the blood volume. If the ultrasound machine is not available, a 10 mL/kg filling test over 15–60 min can be performed.

As a quality process, the revised protocol, including training in echocardiographic evaluation of hemodynamics, will be evaluated after a washout period of 6 months. The results of this new evaluation will help improve the overall management of hemodynamic disorders in neonates born prematurely between 26 and 31 completed weeks of gestation in our unit.

## Figures and Tables

**Table 1:** Main demographic and baseline characteristics.

Characteristic	Whole Cohort (n = 68)	Compliance with the Protocol (n = 8)	Non-Compliance with the Protocol (n = 60)	*p*-Value
**Pregnancy**				
Infectious risk factor	45 (66.1)	4 (50)	41 (68.3)	0.303
Gestational diabetes	4 (5.9)	0 (0)	4 (6.6)	0.452
Pre-eclampsia	13 (19.1)	1 (12.5)	12 (40)	0.612
Complete fetal maturation	49 (73.1)	6 (75)	43 (71)	0.733
Maternal hypotensive treatment	14 (20.6)	1 (12.5)	13 (21.6)	0.547
Magnesium sulfate therapy	9 (13.2)	1 (12.5)	7 (11.6)	0.948
Maternal smoking	19 (27.9)	2 (25)	17 (28.3)	0.844
**Birth**				
Sex				0.964
Male	42 (61.8)	5 (62.5)	37 (61.7)	
Female	26 (38.2)	3 (37.5)	23 (38.3)	
Gestation age, weeks	28 (27–29)	27 (27–29)	28 (27–28.25)	0.977
Spontaneous prematurity	25 (36.8)	4 (50)	21 (35)	0.408
Induced prematurity	43 (63.2)	4 (50)	39 (65)	
Vaginal delivery	37 (54.4)	3 (37.5)	34 (56.7)	0.307
Caesarian	31 (45.6)	5 (62.5)	26 (43.3)	
Apgar at 1 min of life	3 (2–5)	4 (5.5–8)	3 (2–5)	0.38
Apgar at 5 min of life	6 (5–7)	6.5 (5.5–8)	6 (5–7)	0.363
Birth weight, g	965 (839–1200)	1000 (887–1070)	955 (844.5–1234)	0.924
Birth weight	−0.300	0.384	0.325	0.648
Z score	(−1.007–0.206)	(−1.107–0.059)	(−1.054–0,294)	
pH at birth	7.32 (7.26–7.35)	7.33 (7.29–7.36)	7.315 (7.26–7.35)	0.292
Lactate at umbilical cord, mmol/L	2.8 (2.35–4.05)	2.9 (2.8- 3.7)	2.9 (2.2–4.2)	0.947
Diuresis in DR	17 (25)	1 (21.5)	16 (26.7)	0.384
Midazolam in DR	48 (72.7)	5 (62.5)	43 (71.7)	0.488
**Care in NICU**				
Hemoglobin, g/dL	15.2	14.85	15.2	0.376
	(14.2–16.5)	(13.3–15.7)	(14.2–16.5)	
Antibiotics	47 (69.1)	4 (50)	43 (71.7)	0.213
Sedative therapy	65 (95.5)	7(87.5)	58 (96.6)	0.092

Values are given as median (IQR) or n (%). IQR = interquartile range. DR = delivery room.

**Table 2:** Multivariate analysis.

	Regression Coefficient	CI Interval	*p*
Factors involved in non-compliance (*p* < 0.001)			
Echocardiography before management	−0.297	−0.442; −0.153	0.001
Filling duration	−0.240	−0.379; −0.100	0.001
Indication of care	−0.155	−0.341; 0.031	0.100
Dosing of fluid infusion	−0.002	−0.387; 0.383	0.993

CI: Confidence interval.

## Data Availability

For data-sharing inquiries, please contact the corresponding author.

## References

[1] Inserm (2023). La Science Pour la Santé: Prématurité. https://www.inserm.fr/dossier/prematurite/.

[2] Blencowe H., Krasevec J., De Onis M., Black R.E., An X., Stevens G.A., Borghi E., Hayashi C., Estevez D., Cegolon L. (2019). National, regional and worldwide estimates of preterm birthweight in 2015, with trends from 2000: A systematic analysis. Lancet Glob. Health.

[3] Perin J., Mulick A., Yeung D., Villavicencio F., Lopez G., Strong K.L., Prieto-Merino D., Cousens S., Black R.E., Liu L. (2022). Global, regional, and national causes of under-5 mortality in 2000–19: An updated systematic analysis with implications for the Sustainable Development Goals. Lancet Child Adolesc. Health.

[4] Jarreau P.-H. (2016). Pathologies hémodynamiques et cardiovasculaire. Réanimation et Soins Intensifs en Néonatologie.

[5] Morton S.U., Brodsky D. (2016). Fetal Physiology and the Transition to Extrauterine Life. Clin. Perinatol..

[6] Levy M. (2020). Cardiologie Pédiatrique Pratique.

[7] Shead S.L. (2015). Pathophysiology of the Cardiovascular System and Neonatal Hypotension. Neonatal Netw..

[8] Schwarz C.E., Dempsey E.M. (2020). Management of Neonatal Hypotension and Shock. Semin. Fetal Neonatal Med..

[9] Cayabyab R., McLean C.W., Seri I. (2009). Definition of hypotension and assessment of hemodynamics in the preterm neonate. J. Perinatol..

[10] Ng P.C. (2016). Adrenocortical insufficiency and refractory hypotension in preterm infants. Arch. Dis. Child. Fetal Neonatal Ed..

[11] Rameshbabu M., Sundaram V., Sachdeva N., Walia R., Saini S.S., Dutta S. (2018). Association between plasma cortisol and death or vasopressor refractory hypotension in preterm neonates: A prospective, cohort study. J. Perinatol..

[12] Song Y.H., Lee J.A., Choi B.M., Lim J.W. (2021). Risk factors and prognosis in very low birth weight infants treated for hypotension during the first postnatal week from the Korean Neonatal Network. PLoS ONE.

[13] Alderliesten T., Lemmers P.M., van Haastert I.C., de Vries L.S., Bonestroo H.J., Baerts W., van Bel F. (2014). Hypotension in Preterm Neonates: Low Blood Pressure Alone Does Not Affect Neurodevelopmental Outcome. J. Pediatr..

[14] Carrapato M.R.G., Andrade T., Caldeira T. (2019). Hypotension in small preterms: What does it mean?. J. Matern. Fetal Neonatal Med..

[15] Kharrat A., McNamara P.J., Weisz D., Jain A. (2019). Merits and perils of targeted neonatal Echocardiography-Based hemodynamic research: A position statement. Physiol. Pharmacol..

[16] Gupta S., Donn S.M. (2020). Assessment of neonatal perfusion. Semin. Fetal Neonatal Med..

[17] Kluckow M. (2014). Use of ultrasound in the haemodynamic assessment of the sick neonate. Arch. Dis. Child. Fetal Neonatal Ed..

[18] Kluckow M., Seri I., Evans N. (2008). Echocardiography and the Neonatologist. Pediatr. Cardiol..

[19] Sushko K., Al-Rawahi N., Watterberg K., Anker J.V.D., Litalien C., Lacroix J., Razak A., Samiee-Zafarghandy S. (2021). Efficacy and safety of Low-Dose versus High-Dose hydrocortisone to treat hypotension in neonates: A protocol for a systematic review and Meta-Analysis. BMJ Paediatr.

[20] Mullaly R., El-Khuffash A.F. (2024). Haemodynamic assessment and management of hypotension in the preterm. Arch. Dis. Child. Fetal..

[21] Pugnaloni F., De Rose D.U., Kipfmueller F., Patel N., Ronchetti M.P., Dotta A., Bagolan P., Capolupo I., Auriti C. (2024). Assessment of hemodynamic dysfunction in septic newborns by functional echocardiography: A systematic review. Pediatr Res..

[22] Bark B.P., Persson J., Grände P.O. (2013). Importance of the Infusion Rate for the Plasma Expanding Effect of 5% Albumin, 6% HES 130/0.4, 4% Gelatin, and 0.9% NaCl in the Septic Rat. Crit. Care Med..

[23] Weaver L.J., Travers C.P., Ambalavanan N., Askenazi D. (2023). Neonatal fluid overload—Ignorance is no longer bliss. Pediatr. Nephrol..

[24] Binder-Heschl C., Urlesberger B., Schwaberger B., Koestenberger M., Pichler G. (2016). Borderline hypotension: How does it influence cerebral regional tissue oxygenation in preterm infants?. J. Matern. Fetal Neonatal.

[25] Grace E., Keir A.K. (2020). Fluid Therapy. Clin. Perinatol..

